# Comparative analyses of *Mikania* (Asteraceae: Eupatorieae) plastomes and impact of data partitioning and inference methods on phylogenetic relationships

**DOI:** 10.1038/s41598-021-92727-6

**Published:** 2021-06-24

**Authors:** Verônica A. Thode, Caetano T. Oliveira, Benoît Loeuille, Carolina M. Siniscalchi, José R. Pirani

**Affiliations:** 1 Instituto de Biociências, Universidade Federal do Rio Grande do Sul, Avenida Bento Gonçalves, Porto Alegre, Rio Grande do Sul 91501-970 Brazil; 2 Sítio Roberto Burle Marx, Instituto do Patrimônio Histórico e Artístico Nacional, Estrada Roberto Burle Marx, 2019, Barra de Guaratiba, Rio de Janeiro, Rio de Janeiro 23020-240 Brazil; 3 Departamento de Botânica, Centro de Biociências, Universidade Federal de Pernambuco, Avenida Professor Moraes Rego, 1235, Recife, Pernambuco 50670-901 Brazil; 4 Department of Biological Sciences, Mississippi State University, 295 Lee Blvd, Mississippi State, Mississippi, MS 39762 USA; 5 Departamento de Botânica, Instituto de Biociências, Universidade de São Paulo, Rua do Matão, Tv. 14, São Paulo, São Paulo 05508-090 Brazil

**Keywords:** Plant genetics, Plant sciences, Plant evolution

## Abstract

We assembled new plastomes of 19 species of *Mikania* and of *Ageratina fastigiata*, *Litothamnus nitidus,* and *Stevia collina*, all belonging to tribe Eupatorieae (Asteraceae). We analyzed the structure and content of the assembled plastomes and used the newly generated sequences to infer phylogenetic relationships and study the effects of different data partitions and inference methods on the topologies. Most phylogenetic studies with plastomes ignore that processes like recombination and biparental inheritance can occur in this organelle, using the whole genome as a single locus. Our study sought to compare this approach with multispecies coalescent methods that assume that different parts of the genome evolve at different rates. We found that the overall gene content, structure, and orientation are very conserved in all plastomes of the studied species. As observed in other Asteraceae, the 22 plastomes assembled here contain two nested inversions in the LSC region. The plastomes show similar length and the same gene content. The two most variable regions within *Mikania* are *rpl32-ndhF* and *rpl16-rps3*, while the three genes with the highest percentage of variable sites are *ycf1*, *rpoA*, and *psbT*. We generated six phylogenetic trees using concatenated maximum likelihood and multispecies coalescent methods and three data partitions: coding and non-coding sequences and both combined. All trees strongly support that the sampled *Mikania* species form a monophyletic group, which is further subdivided into three clades. The internal relationships within each clade are sensitive to the data partitioning and inference methods employed. The trees resulting from concatenated analysis are more similar among each other than to the correspondent tree generated with the same data partition but a different method. The multispecies coalescent analysis indicate a high level of incongruence between species and gene trees. The lack of resolution and congruence among trees can be explained by the sparse sampling (~ 0.45% of the currently accepted species) and by the low number of informative characters present in the sequences. Our study sheds light into the impact of data partitioning and methods over phylogenetic resolution and brings relevant information for the study of *Mikania* diversity and evolution, as well as for the Asteraceae family as a whole.

## Introduction

Chloroplasts are organelles linked to photosynthesis, which have many essential functions in plants, such as carbon fixation and biosynthesis of starch, fatty acids, amino acids, and pigments^1,2^. The chloroplast genome (plastome) in angiosperms usually has a circular shape, with 120 to 180 kb in size, divided in four main regions: two Inverted Repeat (IR) regions, one Large Single Copy (LSC), and one Small Single Copy (SSC) region^3^. Plastome gene composition and order are generally conserved among land plants^4,5^, but recent studies have documented that variation at many levels can occur^6,7^. It has been widely accepted that plastomes are uniparentally inherited and do not present recombination, with the whole genome frequently being interpreted as a single locus in phylogenetic analysis, implying in all genes evolving concertedly^8^. However, in the last decade, evidence has accumulated that this organelle can be biparentally inherited, copies with different sequences can occur^9^ and that different portions of the genome can evolve at different paces^10^. Recent studies recommend analyzing plastome genes individually in phylogenetic inferences, through methods like the multispecies coalescent, which also accounts for possible incongruence between gene trees and species trees^10,11^.

*Mikania* Willd. is the most diverse genus within the tribe Eupatorieae and the largest genus of climbing plants in Asteraceae, with around 450 species^12,13^. It has a pantropical distribution, mainly neotropical, with most of the diversity concentrated in South America^12,13^. The large number of species makes carrying out taxonomic revisions and molecular studies for the genus difficult^14^. Nevertheless, *Mikania* is easily morphologically recognized by its four-flowered heads surrounded by four involucral bracts; its circumscription has been indisputable since its description in 1742^15^. A reevaluation of the current infrageneric classification of the genus is needed^16^, but the lack of broadly sampled phylogenies prevents the elaboration of a classification based on evolutionary relationships and monophyletic groups^15^. Several regional taxonomic studies of *Mikania* (e.g.^17–19^) are available, but large-scale taxonomic treatments are still a challenge. Some taxa, such as *Mikania glomerata* Spreng. and *M. laevigata* Sch.Bip. ex Baker, have known pharmacological uses, especially in the treatment of respiratory diseases^20^. Consequently, the genus is well represented in phytochemical studies, which have been conducted with approximately 12% of all *Mikania* species^20^. The main chemical compounds linked to pharmacological activities, which are found in different parts of *Mikania* plants, are coumarins and derivatives, sesquiterpenes, sesquiterpenes lactones, diterpenes, phytosterols/terpenoids, and flavonoids^20^. Some species are considered invasive, including some widespread weeds, such as *Mikania micrantha* Kunth^16^.

The first attempt to investigate phylogenetic relationships within *Mikania* with molecular data was based on AFLP markers, the intronic region of the plastid gene *rps16*, and ribosomal ITS and ETS, but included only representatives of generic sections proposed for Brazilian species of *Mikania*^21^. More molecular studies are needed to evaluate infrageneric limits within this genus, as well as to explore its morphological and chemical evolution, biogeographic history, and diversification. The only genomic resources reported in the literature for the genus are the complete plastome (NC031833.1^22^) and the chromosome-scale genome^23^ of *Mikania micrantha*, an invasive plant well known for causing significant damage to natural ecosystems and crops in several parts of the world. For the tribe Eupatorieae as a whole, which has ~ 180 genera and 2200 species^24^, only three other plastomes were published to date, besides *M. micrantha*: i.e., *Ageratina adenophora* (Spreng.) R.M.King & H.Rob. (NC015621^25^), *Praxelis clematidea* R.M.King & H.Rob. (NC023833^26^), and *Ageratum conyzoides* L. (MK905238^27^).

The family Asteraceae is one of the most species-rich families of flowering plants, including an impressive morphological and ecological diversity^28^. While the backbone relationships within the family have recently become clearer, internal relationships at tribal and generic levels still need a lot of attention^28,29^. Although many Asteraceae plastomes were recently published, most studies characterize the plastome of a single taxon and focus in comparative genomic analyses at higher taxonomic levels (e.g.^22,25–27,30,31^). These studies are important to improve the understanding of plastome variation in the family as a whole^27,31^ and to provide more information on their phylogenetic utility. Yet, larger infrageneric samplings are essential to explore evolution and phylogenetic relationships in Asteraceae at lower taxonomic levels (e.g.^32,33^).

In this study, we sequenced new complete plastomes of 19 species of *Mikania* representative of the morphological diversity of the genus. The sampled taxa are: *M. additicia* B.L.Rob*.*, *M. brevifaucia* W.C.Holmes & McDaniel, *M. burchelii* Baker, *M. decora* Poepp., *M. decumbens* Malme, *M. fasciculata* C.T.Oliveira & Pirani, *M. glomerata* Spreng., *M. haenkeana* DC.*, M. lehmanii* Hieron., *M. neurocaula* DC., *M. oblongifolia* DC., *M. obtusata* DC., *M. parvifolia* Baker, *M. purpurascens* (Baker) R.M.King & H.Rob., *M. salviifolia* Gardner, *M. smaragdina* Dusén ex Malme, *M. sylvatica* Klatt., *M. ternata* (Vell.) B.L.Rob., and *M. triangularis* Baker (Table 1). The plastomes of three species from other genera of Eupatorieae, namely *Ageratina fastigiata* (Kunth) R.M.King & H.Rob., *Litothamnus nitidus* (DC.) W.C.Holmes, and *Stevia collina* Gardner, were also sequenced and used as outgroups. The previously published plastome of *Mikania micrantha* (NC031833.1^22^) was included in the analyses as well. This study aims to characterize and compare the plastomes within *Mikania* and among closely related genera within tribe Eupatorieae to improve our understanding about the evolution of this genome and investigate different methods of phylogenetic reconstruction with this dataset. More specifically, we: (i) sequenced, assembled, and characterized the overall plastome structure; (ii) performed comparative genomic analyses within *Mikania,* and among *Mikania* and other Eupatorieae genera; (iii) identified putative repeated regions; and (iv) investigated phylogenetic relationships using both concatenation and multispecies coalescent methods with different data partitions.

**Table 1:** Summary of the plastomes sequenced in this study.

Species	Voucher	GenBank (accession)	Plastome length (bp)	LSC length (bp)	IR length (bp)	SSC length (bp)	Inv1	Inv2
*Mikania additicia* B.L.Rob.	Oliveira 822 (SPF)	MT793849	151,983	83,827	24,927	18,302	22,358	3250
*Mikania brevifaucia* W.C.Holmes & McDaniel	Oliveira 903 (SPF)	MT793850	152,161	83,868	24,980	18,333	22,343	3252
*Mikania burchelii* Baker	Oliveira 706 (SPF)	MT793851	151,829	83,767	24,877	18,308	22,362	3273
*Mikania decora* Poepp. & Endl.	Oliveira 986 (SPF)	MT793834	151,914	83,659	24,964	18,327	22,374	3276
*Mikania decumbens* Malme	Oliveira 783 (SPF)	MT793835	152,056	83,772	24,998	18,288	22,337	3266
*Mikania fasciculata* C.T.Oliveira & Pirani	Oliveira 977 (SPF)	MT793852	152,070	83,747	24,996	18,331	22,307	3249
*Mikania glomerata* Spreng.	Oliveira 917 (SPF)	MT793836	151,773	83,675	24,938	18,222	22,374	3250
*Mikania haenkeana* DC.	Oliveira 897 (SPF)	MT793837	151,865	83,717	24,911	18,326	22,340	3252
*Mikania lehmannii* Hieron.	Oliveira 891 (SPF)	MT793838	152,062	83,878	24,960	18,264	22,369	3250
*Mikania neurocaula* DC.	Fernandes 59 (BHCB)	MT793839	152,045	83,764	24,995	18,291	22,379	3266
*Mikania oblongifolia* DC.	Olivera 966 (SPF)	MT793840	151,845	83,527	24,998	18,322	22,365	3267
*Mikania obtusata* DC.	Oliveira 809 (SPF)	MT793841	152,106	83,817	25,003	18,283	22,406	3266
*Mikania parvifolia* Baker	Fernandes 128 (BHCB)	MT793842	152,023	83,725	24,992	18,314	22,386	3261
*Mikania purpurascens* (Baker) R.M.King & H.Rob.	Oliveira 974 (SPF)	MT793853	152,037	83,746	25,005	18,281	22,337	3249
*Mikania salviifolia* Gardner	Oliveira 813 (SPF)	MT793843	152,229	83,870	25,014	18,331	22,211	3240
*Mikania smaragdina* Dusén ex Malme	Oliveira 798 (SPF)	MT793854	152,020	83,745	24,975	18,325	22,384	3251
*Mikania sylvatica* Klatt.	Oliveira 981 (SPF)	MT793855	152,099	83,808	24,972	18,347	22,363	3257
*Mikania ternata* (Vell.) B.L.Rob.	Oliveira 806 (SPF)	MT793844	151,957	83,791	24,981	18,204	22,324	3250
*Mikania triangularis* Baker	Oliveira 916 (SPF)	MT793845	151,956	83,789	25,002	18,163	22,368	3252
**Outgroups**								
*Ageratina fastigiata* (Kunth) R.M.King & H.Rob.	Oliveira 980 (SPF)	MT793847	152,359	84,009	25,022	18,306	22,423	3,304
*Litothamnus nitidus* (DC.) W.C.Holmes	Oliveira 971 (SPF)	MT793848	151,517	82,844	25,046	18,581	22,326	3223
*Stevia collina* Gardner	Oliveira 769 (SPF)	MT793846	151,248	83,386	24,791	18,280	22,303	3280

## Results

### Plastome assembly and characterization

Approximately 1–2 GB of data and 10,488,036–20,546,020 paired-end raw reads for each plastome were obtained. The 19 *Mikania* plastomes range in length from 151,773 (*M. glomerata*) to 152,229 bp (*M. salviifolia*) (Table 1, Supplementary Fig. S1). All assembled plastomes show the general structure found in most angiosperms, divided in four main regions, which in *Mikania* consists of a LSC (83,527–83,878 bp), a SSC (18,163–18,347 bp), and a pair of IR (24,877–25,014 bp) regions (Table 1, Supplementary Fig. S1). As observed in other Asteraceae taxa, the 22 plastomes assembled here contain two inversions in the LSC region^34^: a large inversion (22,211–22,423 bp, *M. salviifolia* and *Ageratina fastigiata*, respectively) including 16 genes from *trnS*^*GCU*^-*trnC*^*GCA*^ to trn*G*^*UCC*^-*trnT*^*GGU*^ and a small inversion (3223–3304 bp, *Litothamnus nitidus* and *A. fastigiata*, respectively) nested within the former, which includes six genes located between *trnS*^*GCU*^*trnC*^*GCA*^ and *trnE*^*UUC*^ (Table 1, Supplementary Fig. S1). All plastomes sequenced in this study encode 113 unique genes, including 79 protein-coding genes (CDS), 17 of which contain introns, 30 tRNA genes, and four rRNA genes (Table 2). The plastomes of *Mikania* and the other three Eupatorieae have identical structure and order. The boundaries between the four main plastome regions are very conserved within *Mikania* species and among the three Eupatorieae genera sampled here: the LSC/IRb border is within *rps19*, the IRb/SSC is within *ycf1*, the SSC/IRa is between *ndhF* and a partial *ycf1* (*ψycf1*), and the IRa/LSC is between a truncated *rps19* (*†rps19*) and *trnH*^*GUG*^ (Supplementary Figs. S1, S2).

**Table 2:** Genes encoded by the *Mikania* species, *Ageratina fastigiata*, *Litothamnus nitidus,* and *Stevia collina* plastomes.

Gene function	Gene type	Gene
Self-replication	Ribossomal RNA genes	*rrn4.5*^c^*, rrn5*^c^*, rrn16*^c^*, rrn23*^c^
	Transfer RNA genes	*trnA-UGC*^a,c^*, trnC-GCA, trnD-GUC, trnE-UUC, trnF-GAA, trnfM-CAU, trnG-GCC, trnG-UCC*^a^*, trnH-GUG, trnI-CAU*^c^*, trnI-GAU*^a,c^*, trnK-UUU*^a^*, trnL-CAA*^c^*, trnL-UAA*^a^*, trnL-UAG, trnM-CAU, trnN-GUU*^c^*, trnP-UGG, trnQ-UUG, trnR-ACG, trnR-UCU*^c^*, trnS-GCU, trnS-GGA, trnS-UGA, trnT-GGU, trnT-UGU, trnV-GAC*^c^*, trnV-UAC*^a^*, trnW-CCA, trnY-GUA*
	Small ribosomal subunit	*rps2, rps3, rps4, rps7*^c^*, rps8, rps11, rps12*^b,c^*, rps14, rps15, rps16*^a^*, rps18, rps19*^d^
	Large ribosomal subunit	*rpl2*^a,c^*, rpl14, rpl16, rpl20, rpl22, rpl23*^a,c^*, rpl32, rpl33, rpl36*
	RNA polymerase subunits	*rpoA, rpoB, rpoC1*^a^*, rpoC2*
Photosynthesis	Photosystem I	*psaA, psaB, psaC, psaI, psaJ, ycf3*^b^*, ycf4*
	Photosystem II	*psbA, psbB, psbC, psbD, psbE, psbF, psbH, psbI, psbJ, psbK, psbL, psbM, psbN, psbT, psbZ*
	NADH-dehydrogenase	*ndhA*^a^*, ndhB*^a,c^*, ndhC, ndhD, ndhE, ndhF, ndhG, ndhH, ndhI, ndhJ, ndhK*
	Cytochrome b6/f complex	*petA, petB*^a^*, petD*^a^*, petG, petL, petN*
	ATP synthase	*atpA, atpB, atpE, atpF*^a^*, atpH, atpI*
	Rubisco	*rbcL*
Other genes	Translational initiator factor	*infA*
	Maturase	*matK*
	Protease	*clpP*^b^
	Envelope membrane protein	*cemA*
	Subunit of Acetil-CoA-carboxylase	*accD*
	c-type cytochrome synthesis	*ccsA*
Unknown function	Conserved open read frames	*ycf1*^d^*, ycf2*^c^*, ycf15*^c^

^a^Gene with one intron.

^b^Gene with two introns.

^c^Gene duplicated.

^d^Gene partially duplicated.

### Variable regions across plastomes

**Figure 1:**
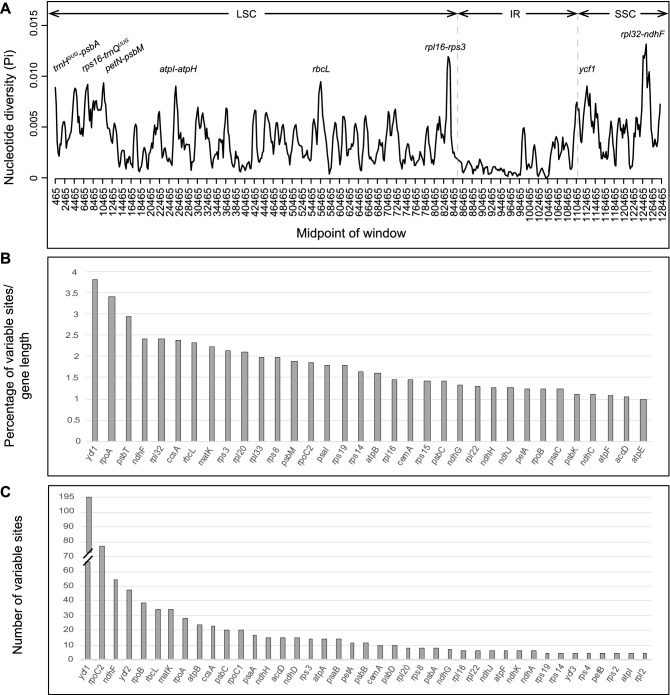
(**A**) Sliding window analysis of the chloroplast genomes of 20 *Mikania* plastomes (i.e., 19 sequenced here plus *M. micrantha*, NC031833.1) (window length: 800 bp, step size: 200 bp). X-axis, position of the midpoint of a window; Y-axis, nucleotide diversity (π) in each window. (**B,C**) Most variable protein-coding genes within the 20 *Mikania* plastomes. (**B**) Genes with up to 1% of variable sites. (**C**) Genes with up to five variable sites.

Pairwise comparison of divergent regions performed in mVISTA within three selected *Mikania* plastomes and between the Eupatorieae genera sequenced here reveals low intra-generic sequence divergence within *Mikania* and higher sequence variation among genera and in noncoding regions, except for the *ycf1* gene (Supplementary Fig. S3). The nucleotide variability (π) values within 800 bp across the plastomes range from 0 to 0.013, with a mean value of 0.0036. We identified only two regions with π > 0.01 (*rpl32-ndhF* and *rpl16-rps3*) and six regions with π values around 0.009 (*rbcL*, *ycf1*, *petN-psbM*, *rps16-trnQ*^*UUG*^, *trnH*^*GUG*^*-psbA*, *atpI-atpH*) (Fig. 1A).

In alignments of the 19 complete *Mikania* plastomes assembled here, the noncoding regions are more variable (i.e., 3.15% of the intergenic regions and 2.24% of the introns) than the coding regions (i.e., 1.34% of the protein-coding genes; Table 3). Among the 79 protein-coding genes, the ten genes with the highest percentage of variable sites are: *ycf1* (3.83%), *rpoA* (3.43%), *psbT* (2.94%), *ndhF* (2.43%), *rpl32* (2.42%), *ccsA* (2.37%), *rbcL* (2.33%), *matK* (2.24%), *rps3* (2.13%), and *rpl20* (2.1%) (Fig. 1B,C; Supplementary Tables S1, S2).

**Table 3:** Summary of datasets including only the 19 *Mikania* plastomes including length, number of variable sites (Var. sites), percentage of variable sites (Var. sites %), parsimony informative sites (Pi sites), and percentage of GC content (GC%).

Dataset	Length (bp)	Var. sites	Var. sites %	Pi sites	GC%
Plastomes (LSC/IR/SSC)	128,486	2966	2.31	1000	36.5
79 genes	67,822	911	1.34	288	37.8
Intergenic regions	62,897	1981	3.15	559	35.2
Introns	13,637	306	2.24	88	34

### Analyses of SSR and tandem repeats

In the 19 *Mikania* plastomes, the total number of SSRs range from 34 to 44 SSRs, while 51, 49, and 38 SSRs are recovered in *A. fastigiata*, *L. nitidus,* and *S. collina*, respectively (Supplementary Fig. S4A–C). The most abundant SSRs are A or T mononucleotide repeats, which account for 54.3–69% of the total SSRs in *Mikania*, 70.6% in *A. fastigiata*, 53.1% in *L. nitidus,* and 52.6% *S. collina*; G or C repeats, on the other hand, are rare (Supplementary Table S3). Among the total number of SSR motifs in *Mikania*, 20–29 (57.5–69%) are mono-repeats, 4–6 (9.5–14.3%) are di-repeats, 2–5 (5.4–12.5%) are tri-repeats, 5–7 (12.2–17.6%) are tetra-repeats, 0–1 (0–2.3%) is penta-repeat, and 0–1 (0–2.9%) is hexa-repeat (Supplementary Fig. S4B, Supplementary Table S3). Furthermore, most of the SSRs in the *Mikania* species are located in the LSC region (70.3–83.8%), while the IR regions include between 0 and 12.2% of the SSRs, and the SSC region includes between 9.1 and 29.7%. Yet, the relative density of SSRs in the LSC is somewhat similar to that found in the SSC when considering the size of each region (Supplementary Fig. S4A–C, Supplementary Table S3). In the three other Eupatorieae genera sequenced here, 73.5–74.5% of the SSRs are located in the LSC, 2–11.8% in the IRs, and 13.7–24.5% in the SSC region (Supplementary Fig. S4A–C, Supplementary Table S3).

We also used REPuter to identify tandem repeat sequences longer than 30 bp in the plastomes sequenced here. In all 22 plastomes, repeats with 30–33 bp are the most common. Most repeats are found in the LSC, a few in the IRs, and none in the SSC (Supplementary Fig. S4D,E, Supplementary Table S4).The total number of repeats in *Mikania* range between 17 and 45, with maximum sizes of 48 bp in all *Mikania* species (Supplementary Fig. S4D,E, Supplementary Table S4). The *Mikania* plastomes contain 8–23 forward repeats, 6–14 palindrome repeats, 0–13 reverse repeats, with complement repeats being rare, 0–2 (Supplementary Table S4). The total number of repeats in *A. fastigiata* is 31 bp, while in *L. nitidus* and *S. collina* is 17 bp. The maximum repeat size in *A. fastigiata* is 46 bp, in *L. nitidus* is 58 bp, while in *S. collina* is 48 bp (Supplementary Fig. S4D,E, Supplementary Table S4).

### Phylogenetic relationships of twenty *Mikania* species

**Figure 2:**
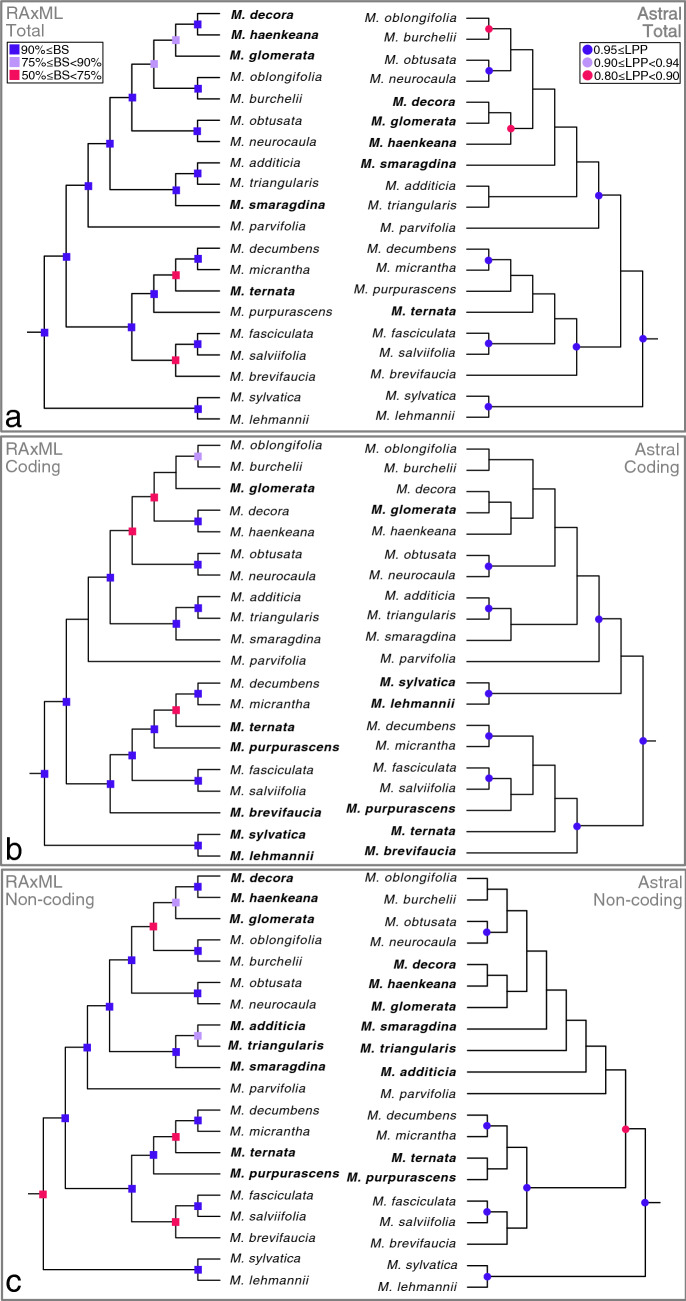
Phylogenetic analyses of *Mikania* using different data partitions and inference methods. RAxML refers to concatenate maximum likelihood analyses and Astral refers to multispecies coalescent inference. Support values are color-coded and nodes without symbols correspond to unsupported branches (BS < 50%, LPP < 0.8). (**a**) Analyses carried out with the whole plastome sequence with one of the IRs removed. (**b**) Analyses conducted only with the coding regions (CDS) of the plastome. (**c**) Analyses carried out only with non-coding regions of the plastome (intergenic regions and introns).

**Figure 3:**
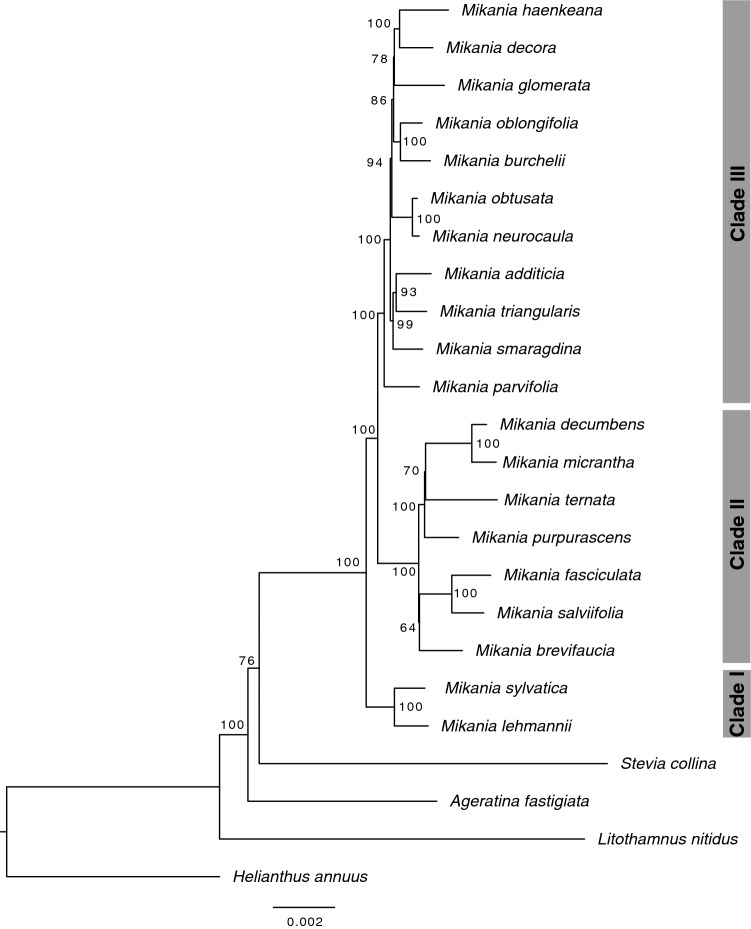
Representative topology of phylogenetic relationships in *Mikania*, showing three main clades. This tree represents the concatenated maximum likelihood analysis conducted with the whole plastome sequence with one of the IRs removed. Bootstrap support values shown in each node.

**Figure 4:**
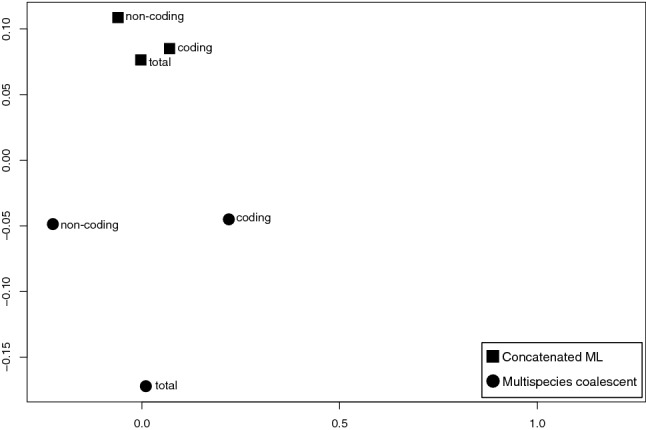
Multidimensional scaling of adjusted Robinson-Foulds values calculated from the pairwise comparison of all rooted trees. Coding: analyses conducted only with the coding regions (CDS). Non-coding: analyses conducted with non-coding regions (intergenic regions and introns). Total: whole plastome sequence with one IR removed.

**Figure 5:**
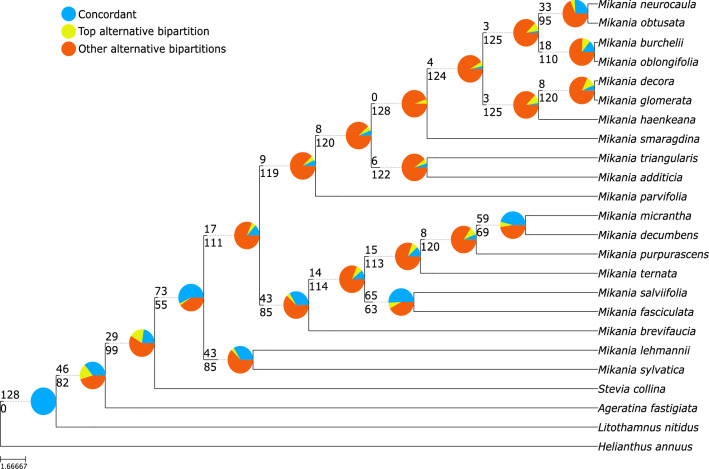
Gene tree discordance analysis conducted with the total dataset and multispecies coalescent inference (Astral total). The number above the branch indicates the number of concordant gene trees and the one below the number of conflicting gene trees. Pie charts indicate the proportion of gene trees supporting that clade (blue), the proportion that supports the main alternative topology for that clade (yellow), and the proportion that supports all other topologies (orange).

Phylogenetic analyses using two different methods, concatenated maximum likelihood and multispecies coalescent, and three datasets (only coding regions, only non-coding regions and both combined) generated six different topologies with different degrees of support (Fig. 2). *Ageratina fastigiata*, *Litothamnus nitidus, Stevia collina*, and *Helianthus annuus* (NC007977; Heliantheae) were used as outgroups and the trees were rooted using *H. annuus* (Fig. 3)*. Mikania* emerges as monophyletic and all trees present three generally well-supported main clades (bootstrap support (BS) ≥ 90%, local posterior probabilities (LPP) ≥ 0.95) containing the same species: Clade I (*Mikania sylvatica* and *M. lehmanii*), Clade II (*M. brevifaucia, M. salviifolia, M. fasciculata, M. purpurascens, M. ternata, M. micrantha, M. decumbens*) and Clade III (*M. parvifolia, M. smaragdina, M. triangularis, M. additicia, M. obtusata, M. neurocaula, M. burchelii, M. oblongifolia, M. glomerata, M. haenkeana, M. decora*). Within each clade, the relationships between some species pairs are stable, but the position of some taxa (e.g., *M. smaragdina, M. ternata)* consistently change, especially in Clade III (Fig. 2). The adjusted Robinson-Foulds distances fitted in a multidimensional scaling model show that topologies are considerably different among themselves, especially the three coalescent trees (Fig. 4, Supplementary Table S5). Although not directly comparable, support values are generally higher in the concatenated analyses (BS) than in the multispecies coalescent analyses (LPP), and in the total dataset in comparison with the coding-only or non-coding-only datasets (Fig. 2). Gene discordance analyses ran with the coalescent trees show a high level of incongruence between the species tree and the gene trees, especially in the dataset containing only coding regions (Fig. 5, Supplementary Fig. S5A).

## Discussion

In this study, we assembled 19 complete plastomes of *Mikania* species and of three other species from tribe Eupatorieae (i.e., *Ageratina fastigiata*, *Litothamnus nitidus,* and *Stevia collina*), and conducted phylogenetic analyses with different datasets and inference methods*.* The organization of *Mikania* plastomes is similar across the studied species and to other Asteraceae plastomes. The overall genomic structure among *Mikania* plastomes is very conserved, including similar lengths, boundaries between the SC/IR regions, and number of duplicated genes in the IRs (Tables 1, 2, Supplementary Figs. S1–S3). All plastomes assembled here show the two inversions in the LSC present in most Asteraceae taxa, except for the early diverging tribe Barnadesieae^30,35,36^. These inversions present a very conserved structure, including the same genes and similar sizes, in all of the 22 plastomes reported here (Table 1), as well as when comparing with more distantly related Asteraceae genomes, such as *Helianthus annuus* (NC007977^30^) and *Lactuca sativa* (NC007578^30^). On the other hand, in *Ageratina adenophora*^25^ and *Praxelis clematidea*^26^, these inversions do not start between the *trnS*^*GCU*^ and *trnC*^*GCA*^ genes, as in other Asteraceae taxa, but between the *trnC*^*GCA*^ and *petN* genes^25,26^. We also noticed an inversion within the *ycf1* gene in the SSC region in the *Ageratina adenophora*^25^ and *Praxelis clematidea*^26^ plastomes, which was not observed in the plastomes assembled here, nor in *M. micrantha* (NC031833.1^22^) or *Ageratum conyzoides* (MK905238^27^) (Supplementary Fig. S6).

The gene content found in the 22 plastomes assembled here resembles that found in other Asteraceae genomes^25,31,33^. They encode 113 unique genes, including 79 protein-coding genes (CDS), 30 tRNA genes, and four rRNA genes. All plastomes include 17 intron-containing genes (14 contain one, while three contain two introns; Table 3). Within Eupatorieae, a duplication of the *trnF*^*GAA*^ gene was detected in *Ageratina adenophora*^25^ and *Praxelis clematidea*^26^, which was previously reported for other Asteraceae subfamilies (Carduoideae, Cichorioideae, Asteroideae, and Heliantheae alliance)^37^. The *rpoC1* gene in *Ageratina adenophora*^25^, *Ageratum conyzoides*^27^, and *Praxelis clematidea*^26^ contains two introns, while in all plastomes assembled here it contains only one intron, similarly to other Asteraceae plastomes sequenced to date^25,33^. Previous plastome comparative studies within Heliantheae detected a ~ 450 bp deletion in the *ycf2* gene for some taxa^30,33^, which was not observed in the previously published Eupatorieae plastomes or those newly sequenced here.

The nucleotide variability is relatively low within *Mikania* plastome sequences (mean π value of 0.0036). Yet, another comparative plastome study in Asteraceae, with 36 species of *Aldama* (Heliantheae), found an even lower mean π value, 0.00118^33^. The *rpl32-ndhF* and *rpl16-rps3* intergenic regions are the most variable loci found within *Mikania* plastomes, making them candidate markers for phylogenetic studies at the species level within the genus. Other regions with higher nucleotide variability within *Mikania* are: *rbcL*, *ycf1*, *petN-psbM*, *rps16-trnQ*^*UUG*^, *trnH*^*GUG*^*-psbA*, and *atpI-atpH* (Fig. 4A). The noncoding regions are more variable that the coding regions, as expected^38^ (Supplementary Fig. S3, Supplementary Table S2). Some of the noncoding regions that are variable within *Mikania* have been reported to be likely useful for molecular studies at lower taxonomic levels^39,40^. Considering only the coding regions and the percentage of variable sites, the *ycf1* gene is the most variable (3.83%) followed by *rpoA* (3.43%) (Fig. 4B,C, Supplementary Table S1). The *ycf1* gene is well known as a variable coding region at lower taxonomic levels, including within Asteraceae, and has been used in phylogenetic studies within distinct plant groups^33,41^. In addition, the *rpo* genes (*rpoA*, *rpoB*, *rpoC1*, and *rpoC2*) have been previously reported to be relatively rapidly evolving^42,43^ and divergent within Asteraceae^25,33,41^.

The number of Single Sequence Repeats (SSRs), 34 to 44, identified within *Mikania* plastomes is similar to that reported for other Asteraceae, such as within Heliantheae, where 38–57 SSRs were found in a study with 15 species^33^. In all plastomes assembled here, most SSRs found are mononucleotide repeats (59–61% within *Mikania* and 22–37% in the other genera), followed by tetranucleotide repeats (5–7% within *Mikania* and 57–72% in the other genera). The A or T motifs are the most common SSR repeat, in agreement with other studies^31,44^ (Supplementary Fig. S4A–C, Supplementary Table S3). Dispersed repeats are considered to have important influence in genome structure, size, recombination, and rearrangements^25^. The number of repeats ≥ 30 bp found in the plastomes sequenced in this study range from 17 to 45. The maximum repeat size found within all *Mikania* species was 48 bp (Supplementary Fig. S4D,E, Supplementary Table S4). In *Myripnois dioica* (Pertyeae) 58 repeats ≥ 20 bp were found and the maximum repeat length was the same found for *Mikania* (48 bp)^31^. In *Lactuca sativa* and *Helianthus annuus*, 15 and 33 repeats ≥ 23 bp were found, respectively, of which most were smaller than 40 bp, with only two larger than 90 bp^30^. In *Ageratina denophora*, 59 repeats ≥ 15 bp were found, most ranging between 15 and 50 bp, but repeats > 100 bp were also present^25^.

The phylogenetic analyses performed here sampled only 20 out of ~ 450 *Mikania* species (Fig. 3). Yet, the relationships within this genus were never investigated using complete plastomes and represent an advance in our knowledge of infrageneric evolutionary relationships. The only phylogenetic study of the genus to date^21^ was focused on species delimitation of a few highly variable taxa, such as *M. micrantha* and *M. cordifolia*, based on AFLPs and two nuclear ribosomal markers*.* The divergent sampling, with only four species overlapping between both studies, precludes a proper comparison between the topologies from the previous study and the ones found here. The differences in the genomic compartment used by both studies further hinders a proper comparison, given the frequent occurrence of discrepancies between nuclear and plastidial phylogenies. The comparison among trees obtained with different reconstruction methods and datasets show a scenario of incongruence among topologies, especially in the higher nested clades (Figs. 2, 4). The backbones of most trees show Clade I as sister to a clade formed by Clade II and Clade III, except for the Astral coding tree, which shows Clade II as sister to Clade I and Clade III (Fig. 2B). Clade II presents the same relationships in all three concatenated analyses, while in the coalescent trees the relative positions of some taxa, such as *Mikania ternata* and *M. purpurascens*, change in all trees, but especially when comparing the coding tree with the non-coding and total trees (Fig. 2). The relationships within Clade III are responsible for most of the incongruence among all six trees, as they change in each topology. Some species emerge as sisters in most topologies, such as *M. decora* + *M. haenkeana, M. oblongifolia* + *M. burchelii, M. obtusata* + *M. neurocaula* and *M. additicia* + *M. triangularis.* Similarly, *M. parvifolia* emerges as sister to all other species in Clade III in all analyses, but the relative positions of *M. smaragdina* and *M. glomerata* are variable across all topologies, usually with *M. smaragdina* being close to *M. additicia* and *M. triangularis,* and *M. glomerata* close to *M. decora* and *M. haenkeana* (Fig. 2)*.*

The gene tree discordance analyses (Fig. 5, Supplemental Fig. S5) show strong discordance across the three datasets (total, coding, non-coding), with few gene trees agreeing with the relationships shown in the species tree. The multispecies coalescent has been extensively used in the context of multi-locus phylogenies obtained from target capture data^45^, but few studies have applied it to plastid data, due to the widespread misconceptions about the lack of biparental inheritance and recombination in this organelle^11^. Among recent studies that used the multispecies coalescent in plastid data, three of them refer to higher-level phylogenies, i.e., among Angiosperms^46^, among Rosids^10^ and among tribes of Asteraceae^47^, while one deals with a single genus^48^. Most of these studies found incongruences between concatenated and coalescent analyses, but only two of them presented information about gene tree/species tree discordance^46,47^, both showing wide discordance between the inferred species tree and the gene trees.

Walker et al.^46^ proposes that uninformative genes are one of the reasons for discordance, and this is likely one of the issues in our trees. In the coding dataset, the number of variable sites in each gene across species of *Mikania* varies from 0 to 3.83%, while the non-coding dataset presents a little more variation, from 0.5% to 8.8% (Supplementary Tables S1, S2). The large variation in gene tree topologies, summarized by the gene tree discordance analysis (Fig. 5, Supplementary Fig. S5), leads to weakly resolved gene trees and consequently to poorly supported species trees, as the calculation of local posterior probabilities (LPP) depends on the concordance among the three possible topologies for a determined quartet of branches^49^. The length of each individual locus alignment also influences on the degree of conflict, as shorter loci tend to have less informative sites, contributing to the lack of resolution in gene trees^46^. In our dataset, these two factors seem to be correlated, with most of the shorter alignments presenting very few variable sites (Supplementary Fig. S7, Supplementary Table S1, S2).

Recent studies have shown that in concatenated analysis, a few outlier genes can drive topology inference^45,50^. Our concatenated analyses present topologies more similar to each other than the coalescent topologies, regardless of the dataset (Fig. 4). The concatenated coding tree is more similar to the concatenated total topology (Figs. 2, 4), possibly indicating that one or more specific genes are responsible for defining most of the topology, whereas the three coalescent topologies are all different from each other, due to lack of resolution in individual gene trees. Incomplete lineage sorting (ILS) is usually considered a source of conflict in the multispecies coalescent^45^, and one metric that can be used to assess its occurrence is the normalized quartet score of a coalescent tree, which measures the percentage of quartet trees found in the species tree from all calculated quartet trees^51^. The normalized quartet scores calculated for the three Astral topologies are considered very high (total: ~ 52%, coding: ~ 49%, non-coding: ~ 57%), but considering the sparse sampling in our study, which included ca. 0.45% of *Mikania* species, it is difficult to delimit the occurrence of ILS in relation to the lack of actual sampling. Further studies with more complete sampling could help untangle cases of ILS and lack of resolution due to uninformative genes, by also increasing the likelihood of sequence variation.

Although applying the multispecies coalescent methods to chloroplast sequences makes sense biologically, due to the possibility of evolutionary process that could lead to different parts of the genome evolving in different rates, in practice the results tend to be confounding, as seen here and in previous studies^10,46^. The causes of plastome conflict are still poorly understood^46^, and in lower-level phylogenies, as the case presented here, it might be hard to untangle sources of conflict inherent to plastome biology from lack of sequence variability due to rapid radiations over short evolutionary times. In *Mikania,* where the phylogenetic relationships are poorly known, especially in relation to the nuclear genome, it is difficult to map out other potential root causes for conflict, such as hybridization, plastome capture, or incomplete lineage sorting. An expanded sampling, both in terms of species and genome compartment (e.g., adding nuclear markers), could bring a clearer picture of the evolutionary relationships in the genus and of other biological factors that might impact phylogenetic reconstructions in *Mikania*.

## Material and methods

### Sampling, DNA preparation, sequencing, plastome assembly, and annotation

Whole genomic DNA extraction, Illumina libraries preparation, and NGS sequencing of the 19 *Mikania* and three outgroup species from other Eupatorieae genera (Table 1) follow^33^. Sequencing was performed using Illumina HiSeq 2500 Genome Analyzer (Illumina, San Diego, California, USA) in paired-end mode. We assembled all plastomes using Fast-Plast 1.2.8^52^, with the following software: (i) Trimmomatic 0.32^53^ to remove adaptors and trim low quality reads using the parameters SLIDINGWINDOW:10:20 and MINLEN:40; (ii) Bowtie2 2.1.0^54^ with default parameters to select only chloroplast-like reads using the plastome of *Mikania micrantha* (NC031833.1^22^ as reference; (iii) SPAdes 3.1.0^55^ to assemble the selected reads into contigs with k-mers of 57 and 87, using the “only-assembler” option; (iv) afin (http://bitbucket.org/benine/afin/) to assemble the contigs from the previous step with the complete reads dataset with the following parameters -l 150,50,50, -f 0.1, -d 100,—× 100, -p 20,15,10, and -i 2,1,1. We evaluated plastome coverage in Jellyfish 2.1.3^56^. We annotated the sequences using Geneious 9.1.5^57^, DOGMA^58^, and BLAST^59,60^, with start and stop codons checked manually. We used OGDRAW^61^ to prepare the graphical representation for the resulting plastome. Finally, we analyzed the boundaries between the plastome regions (i.e., LSC/IRb/SSC/IRa) using Geneious and IRscope^62^ (https://irscope.shinyapps.io/irapp/).

### Comparative analyses of the assembled *Mikania* plastomes

We conducted comparative analyses within 20 *Mikania* plastomes (i.e., 19 sequenced here plus *M. micrantha*, NC031833.1) and among *Mikania* and the three outgroup taxa plastomes assembled in this study (i.e., *Ageratina fastigiata*, *Litothamnus nitidus,* and *Stevia collina*). We used MAFFT 7^63^ with the FFT-NS-2 method^64^ to perform the alignment of the complete plastome sequences, with one copy of the IRs manually excluded to avoid data duplication. To search variable regions, we used mVISTA^65^ with Shuffle-LAGAN^66^ with the previously annotated *M. decora* plastome as reference, plus two *Mikania* species and the three outgroup taxa sequenced here. Based on the phylogeny recovered in this study, we selected one species from each of the three main recovered clades of *Mikania* (i.e., *M. decora*, *M. decumbens*, and *M. sylvatica*). We calculated nucleotide variability values (π) within 20 *Mikania* plastomes. We used DnaSP 6.10^67^ to conduct a sliding window analysis with a 200 bp step size and 800 bp window length. The resulting π values were plotted using R^68^. We analyzed the variable sites using MEGA 7^69^ in the alignments of the 20 *Mikania* complete plastomes and of 79 protein-coding genes (Supplementary Table S1) extracted from these genomes. Each gene was extracted from the complete plastome alignment and separately re-aligned in Geneious with the ClustalW plugin^70^ considering codon positions.

### Analyses of repeated regions

We searched for microsatellites or Simple Sequence Repeats (SSRs; i.e.*,* tandemly arranged repeats of short DNA motifs of 1–6 bp in length) and repeated elements using MISA^71^ and REPuter^72^, respectively, in the plastomes of the 19 *Mikania* species and three other Eupatorieae representatives sequenced here. We analyzed SSRs with motifs between 1 and 6 nucleotides and a minimum number of repetition units as follows: 10 for mono-, 5 for di-, and 4 for trinucleotide, and 3 for tetra-, penta-, and hexanucleotide SSRs. We identified repeated elements ≥ 30 bp (forward, palindrome, reverse, and complement) using ≥ 90% of sequence identity and hamming distance = 3.

### Phylogenetic reconstruction

We reconstructed phylogenetic relationships among 20 *Mikania* plastomes (i.e., 19 sequenced here plus *M. micrantha*; NC031833.1) and three species from other Eupatorieae genera assembled in this study plus *Helianthus annuus* (NC007977; Heliantheae) as outgroup. Three concatenated matrices were assembled: one containing the whole plastome sequence with one IR removed (total), one containing only the CDS regions of all 79 protein-coding genes (coding) and one containing all intergenic regions and introns (non-coding). All matrices were aligned using MAFFT 7^63^ using the FFT-NS-2 method^64^. Maximum likelihood reconstructions were carried out in in RAxML 8.2.9^73^ using the GTR + G model with node support assessed by rapid bootstrap (-f a) using 1000 non-parametric bootstrap pseudo-replicates. The multispecies pseudocoalescent model from Astral III^51^ was used to obtain species trees from individual gene trees. Three datasets were used in these analyses: one containing only each individual CDS region from all 79 protein-coding genes (coding), one containing intergenic regions longer than 300 bp (non-coding), and one combining both datasets (total). Character evolution models for each gene matrix were calculated with PartitionFinder v.1.1.0^74–76^, evaluating the GTR + G and GTR + G + I models in the RAxML version with rcluster search option and unlinked branch lengths, using the corrected Akaike Information Criterion to choose between models. Unrooted gene trees were obtained in RAxML 8.2.9, using the rapid bootstrap mode and 100 pseudo-replicates. Branch support was calculated using local posterior probabilities (LPP)^51^.

### Gene tree discordance

Discordance between the species tree and gene trees, expressed as the proportion of gene trees presenting each of the clades found in the species tree, was calculated using phyparts with the thorough conflict analysis options (-a 1)^77^. All species and gene trees were rooted using *Helianthus annuus* as outgroup using the function pxrr in the package phyx^78^. The proportion of gene trees in agreement with the species tree in each node, as well as the proportion of uninformative gene trees or those supporting alternative topologies, were plotted as pie charts at each node of the tree using the phypartspiecharts.py script^79^.

### Topological comparisons

The adjusted Robinson-Foulds (RF) distance was used to calculate the distance among the six topologies. The RF distance was calculated between all pairs of rooted trees using PAUP* v4.0a^80^ and adjusted by the number of nodes in the trees (RFadj = RF/(2n − 6)), resulting in values ranging from 0 to 1. A multidimensional scaling approach was used to observe the level of similarity among the topologies, using the “cmdscale” command in the R package “stats”, and subsequently plotted.

### Data archiving statement

The complete plastome sequence data of the 19 *Mikania* plastomes and that of *Ageratina fastigiata*, *Litothamnus nitidus*, and *Stevia collina* are available in GenBank (NCBI) with the accession numbers MT793834–MT793855.

## Supplementary Information

Supplementary Information.
