# Supplementary material for: Comparative analyses of Mikania (Asteraceae: Eupatorieae) plastomes and impact of data partitioning and inference methods on phylogenetic relationships
Source: Sci Rep. 2021 Jun 24;11:13267. doi: 10.1038/s41598-021-92727-6 (PMC8225666; doi:10.1038/s41598-021-92727-6)
Supplement: Supplementary file 1 — Supplementary Information. [file 41598_2021_92727_MOESM1_ESM.pdf]

Supplementary Material

Comparative analyses of *Mikania* (Asteraceae: Eupatorieae) plastomes and impact of data partitioning and inference methods on phylogenetic relationships

Verônica A. Thode, Caetano T. Oliveira, Benoît Loeuille, Carolina M. Siniscalchi[*] and José R. Pirani[5]

*** Correspondence:** *carol.siniscalchi@gmail.com*

Supplementary Tables

Comparative analyses of *Mikania* (Asteraceae: Eupatorieae) plastomes and impact of data partitioning and inference methods on phylogenetic relationships

Verônica A. Thode, Caetano T. Oliveira, Benoît Loeuille, Carolina M. Siniscalchi* and José R. Pirani[5]

*** Correspondence:** *carol.siniscalchi@gmail.com*

Supplementary Table 1. Summary of the 79 protein-coding gene alignments extracted from 19 *Mikania* species plastomes: alignment length (bp), length of alignment translated in amino acids, number of variable sites, percentage of variable sites, parsimony informative sites (Pi sites), and percentage of GC content (GC%) for the nucleotide alignments.

Gene	Length (bp)	Length translated	Var. sites	% variable	Pi sites	GC %
ndhE	306	102	0	0.00	0	31.4%
petD	486	162	0	0.00	0	39.1%
petG	114	38	0	0.00	0	36.0%
petL	96	32	0	0.00	0	34.4%
petN	90	30	0	0.00	0	41.4%
psaJ	135	45	0	0.00	0	41.5%
psbH	222	74	0	0.00	0	38.3%
psbL	117	39	0	0.00	0	33.3%
psbZ	189	63	0	0.00	0	36.0%
rpl23	282	94	0	0.00	0	39.4%
rps7	468	156	0	0.00	0	40.8%
ndhB	1,533	511	2	0.13	2	37.4%
ndhI	501	167	2	0.40	0	34.8%
psbE	252	84	1	0.40	0	41.7%
atpH	246	82	1	0.41	0	44.7%
infA	234	78	1	0.43	0	35.9%
rps11	411	137	2	0.49	1	46.0%
clpP	592	197	3	0.51	1	42.1%
ycf4	555	185	3	0.54	1	38.2%
rpl14	369	123	2	0.54	0	40.1%
ndhA	1,092	364	6	0.55	1	34.2%
rps12	357	119	2	0.56	0	44.2%
rpl2	825	275	5	0.61	1	43.7%
psaB	2,205	735	14	0.63	7	40.3%
atpI	744	248	5	0.67	1	38.8%
ycf2	6831	2.277	47	0.69	12	37.7%
rps2	711	237	5	0.70	0	37.4%
psaA	2253	751	17	0.75	2	43.1%
psbA	1,062	354	8	0.75	3	41.9%
rps16	267	89	2	0.75	0	37.9%
psbN	132	44	1	0.76	1	42.5%
petB	648	216	5	0.77	0	39.5%
psbB	1527	509	12	0.79	2	43.7%
psbJ	123	41	1	0.81	0	41.1%
rps4	606	202	5	0.83	1	39.8%
psbF	120	40	1	0.83	0	41.6%

Gene	Length (bp)	Length translated	Var. sites	% variable	Pi sites	GC %
ndhK	678	226	6	0.88	3	38.5%
rpl36	114	38	1	0.88	0	35.1%
psbI	111	37	1	0.90	0	36.1%
atpA	1,527	509	14	0.92	5	40.4%
psbD	1,062	354	10	0.94	3	42.6%
rpoC1	2.07	690	20	0.97	3	37.9%
rps18	306	102	3	0.98	2	34.0%
ndhD	1,521	507	15	0.99	3	36.1%
ycf3	507	169	5	0.99	3	39.2%
atpE	402	134	4	1.00	0	40.0%
accD	1,452	484	15	1.03	5	36.3%
atpF	555	185	6	1.08	2	37.7%
ndhC	363	121	4	1.10	0	34.2%
psbK	180	60	2	1.11	1	37.2%
psaC	246	82	3	1.22	1	42.3%
rpoB	3,183	1.061	39	1.23	10	38.4%
petA	963	321	12	1.25	5	40.3%
ndhJ	477	159	6	1.26	2	39.8%
ndhH	1,182	394	15	1.27	4	37.9%
rpl22	465	155	6	1.29	1	34.2%
ndhG	531	177	7	1.32	3	36.0%
psbC	1,422	474	20	1.41	4	43.3%
rps15	279	93	4	1.43	1	32.7%
cemA	690	230	10	1.45	2	32.9%
rpl16	411	137	6	1.46	1	41.9%
atpB	1,497	499	24	1.60	4	42.0%
rps14	303	101	5	1.65	3	41.9%
rps19	279	93	5	1.79	1	33.4%
psaI	111	37	2	1.80	0	36.9%
rpoC2	4,146	1.382	77	1.86	25	38.0%
psbM	105	35	2	1.90	1	28.7%
rps8	405	135	8	1.98	7	36.5%
rpl33	201	67	4	1.99	0	40.3%
rpl20	381	127	8	2.10	3	37.1%
rps3	657	219	14	2.13	11	33.8%
matK	1,515	505	34	2.24	7	33.7%
rbcL	1,458	486	34	2.33	24	43.6%
ccsA	969	323	23	2.37	6	31.6%
rpl32	165	55	4	2.42	2	34.4%
ndhF	2,226	742	54	2.43	20	32.4%
psbT	102	34	3	2.94	3	32.8%

Gene	Length (bp)	Length translated	Var. sites	% variable	Pi sites	GC %
rpoA	816	272	28	3.43	6	36.4%
ycf1	5,088	1.696	195	3.83	65	29.6%

Supplementary Table 2. Summary of the 49 alignments of intergenic regions extracted from 19 *Mikania* species plastomes: alignment length (bp), length of alignment translated in amino acids, number of variable sites, percentage of variable sites, parsimony informative sites (Pi sites), and percentage of GC content (GC%) for the nucleotide alignments.

Intergenic region	Length (bp)	Var. sites	% variable	Pi sites	GC %
trnL-CAA-ycf2	412	2	0.5	2	45.6
ycf2-trnL-CAA	412	2	0.5	2	45.1
trnN-GUU-ycf1	320	2	0.6	0	37.2
ycf1-trnN-GUU	320	2	0.6	0	37.2
ndhB-trnL-CAA	568	4	0.7	2	37.1
trnL-CAA-ndhB	573	4	0.7	2	36.6
trnV-GAC-ycf15	690	6	0.9	3	41.2
ycf15-trnV-GAC	690	6	0.9	3	41.4
rps7-ycf15	1909	20	1	12	38.9
ycf15-rps7	1918	20	1	12	39
trnP-UGG-psaJ	325	5	1.5	1	28.8
trnN-GUU-trnR-ACG	495	8	1.6	1	45.7
trnR-ACG-trnN-GUU	501	8	1.6	1	45.2
psaJ-rpl33	443	8	1.8	1	32.5
trnSUGA-psbZ	339	7	2.1	1	35.5
rbcL-aacD	596	13	2.2	3	30.6
psbE-petL	1358	31	2.3	13	29.5
rpl20-clpP	1058	26	2.5	12	34.6
rps4-trnT-UGU	387	10	2.6	4	24
clpP-psbB	471	14	3	3	30.5
trnTGGU-psbD	1258	38	3	10	31
accD-psaI	809	25	3.1	9	23.7
atpH-atpF	378	12	3.2	6	31.3
psbM-trnDGUC	679	22	3.2	9	34.1
ycf3-trnSGGA	979	32	3.3	14	28.4
atpB-rbcL	849	31	3.7	8	30
psaA-ycf3	802	30	3.7	13	30.7
psbK-psbI	428	16	3.7	10	31.2
rps16-psbK	1478	54	3.7	24	29.5
trnSGCU-trnCGCA	780	31	4	14	29.9
trnSGGA-rps4	379	15	4	4	34.9
atpI-atpH	1230	50	4.1	20	31.1
trnCGCA-petN	874	36	4.1	13	32.5
ndhC-trnV-UAC	1317	56	4.3	25	29.5
ycf4-cemA	924	40	4.3	12	30
matK-rps16	1704	75	4.4	27	29.8
psbZ-trnCGGC	340	15	4.4	7	31.1

ndhI-ndhG	423	19	4.5	6	23.6
trnEUUC-rpoB	955	45	4.7	12	33.5
trnT-UGU-trnL-UAA	595	28	4.7	15	27.5
psbA-matK	648	32	4.9	8	29.2
trnL-UAA-trnF-GAA	348	17	4.9	6	33.8
psaI-ycf4	412	21	5.1	7	34.7
ycf1-rps15	442	23	5.2	5	25.8
trnL-UAG-rpl32	865	52	6	26	24.7
petA-psbJ	868	54	6.2	20	29.7
petN-psbM	590	41	6.9	13	28
rpl32-ndhF	1099	84	7.6	31	24.5
trnH-GUG-psbA	510	45	8.8	27	24.9

Supplementary Table 3. Results from the distribution of SSRs in the plastomes of the 19 *Mikania* species studied here and that of *Ageratina fastigiata*, *Litothamnus nitidus,* and *Stevia collina* (Eupatorieae).

Species	SSRs total	Unit size						Total per region			Repeat type			
		mono	di	tri	tetra	penta	hexa	LSC	IR	SSC	A/T repeats	% of A/T repeats from total	A repeats	T repeats
Mikania additicia	44	28	6	3	6	0	1	36	4	4	26	59.1	12	14
Mikania brevifaucia	40	24	5	3	7	0	1	31	4	5	24	60.0	11	13
Mikania burchelii	36	22	5	2	6	0	1	26	2	8	21	58.3	9	12
Mikania decora	36	22	5	3	5	0	1	27	4	5	21	58.3	11	10
Mikania decumbens	41	27	5	3	5	0	1	32	5	4	26	63.4	10	16
Mikania fasciculata	42	29	4	3	6	0	0	31	2	9	29	69.0	13	16
Mikania glomerata	38	23	5	3	6	0	1	30	2	6	23	60.5	10	13
Mikania haenkeana	38	23	5	3	6	0	1	29	3	6	22	57.9	11	11
Mikania lehmanii	35	21	5	3	5	0	1	27	3	5	19	54.3	9	10
Mikania neurocaula	42	27	5	3	6	0	1	32	3	7	27	64.3	12	15
Mikania oblongifolia	37	22	5	3	6	0	1	28	2	7	22	59.5	11	11
Mikania obtusata	39	24	5	3	6	0	1	30	3	6	24	61.5	11	13
Mikania parvifolia	42	27	5	3	6	0	1	32	4	6	26	61.9	12	14
Mikania purpurescens	43	27	5	3	6	1	1	32	4	7	27	62.8	12	15
Mikania salviifolia	34	20	5	3	6	0	0	25	2	7	20	58.8	9	11
Mikania smaragdina	41	25	5	3	7	0	1	31	3	7	24	58.5	12	12
Mikania sylvatica	37	24	5	2	5	0	1	26	0	11	22	59.5	10	12
Mikania ternata	40	23	5	5	7	0	0	30	4	6	23	57.5	9	14
Mikania triangularis	37	22	5	3	6	0	1	31	0	6	22	59.5	11	11
**Outgroups**														
Ageratina fastigiata	51	37	3	4	6	0	1	38	6	7	36	70.6	15	21
Litothamnus nitidus	49	28	7	3	8	2	1	36	1	12	26	53.1	14	12
Stevia collina	38	22	6	1	6	2	1	28	2	8	20	52.6	12	8

Supplementary Table 4. Results from the REPuter analysis of distribution of repeat sequences in the plastomes of the *Mikania* species studied here plus *Ageratina fastigiata*, *Litothamnus nitidus,* and *Stevia collina*, all belonging to tribe Eupatorieae. F = forward, P = palindrome, and R reverse repeats.

Species	Total	Total repeats per region			Repeat type				Maximum size	Number of repeats per size					
		LSC	IR	SSC	F	P	R	C		30-33bp	34-37bp	38-41bp	42-45bp	46-49bp	>49bp
Mikania additicia	27	23	4	0	8	6	13	0	48	12	7	5	1	2	0
Mikania brevifaucia	21	16	5	0	13	8	0	0	48	12	3	2	2	2	0
Mikania burchelii	21	16	5	0	13	8	0	0	48	12	3	3	1	2	0
Mikania decora	17	13	4	0	10	7	0	0	48	11	2	3	0	1	0
Mikania decumbens	45	40	5	0	23	9	13	0	48	28	9	3	3	2	0
Mikania fasciculata	27	16	5	6	14	9	3	1	48	15	5	2	2	3	0
Mikania glomerata	34	29	5	0	18	9	7	0	48	25	3	3	1	2	0
Mikania haenkeana	19	14	5	0	12	7	0	0	48	11	3	3	1	1	0
Mikania lehmanii	44	39	5	0	21	13	8	2	48	35	3	3	1	2	0
Mikania neurocaula	20	15	5	0	12	8	0	0	48	11	3	3	1	2	0
Mikania oblongifolia	20	15	5	0	12	8	0	0	48	11	3	3	1	2	0
Mikania obtusata	22	17	5	0	13	8	1	0	48	12	4	3	1	2	0
Mikania parvifolia	22	17	5	0	13	8	0	1	48	14	2	3	1	2	0
Mikania purpurescens	35	27	5	3	15	14	4	2	48	24	5	2	2	2	0
Mikania salviifolia	22	17	5	0	14	8	0	0	48	12	2	3	3	2	0
Mikania smaragdina	23	18	5	0	12	10	1	0	48	14	2	4	1	2	0
Mikania sylvatica	20	14	5	1	11	8	1	0	48	12	2	3	1	2	0
Mikania ternata	20	16	4	0	11	9	0	0	48	13	2	2	1	2	0
Mikania triangularis	21	16	5	0	13	8	0	0	48	13	2	3	1	2	0
**Outgroups**															
Ageratina fastigiata	31	15	16	0	14	8	9	0	46	22	3	3	2	1	0
Litothamnus nitidus	17	11	5	1	8	9	0	0	58	11	0	4	0	1	1
Stevia collina.	17	14	3	0	8	9	0	0	48	8	2	6	0	0	1

Supplementary Table 5. Adjusted pairwise Robinson-Foulds distances calculated among all six topologies. Values range from ranges from 0 (similar topologies) to 1 (completely discordant topologies). Astral: multispecies coalescent analyses, RAxML: concatenated maximum likelihood analyses, CDS: coding regions only, IG: non-coding regions only, total: both CDS and IG regions combined.

Topology	**Astral CDS**	**Astral IG**	**Astral Total**	**RAxML CDS**	**RAxML IG**	**RAxML Total**
**Astral CDS**	0	–	–	–	–	–
**Astral IG**	0.45	0	–	–	–	–
**Astral Total**	0.25	0.25	0	–	–	–
**RAxML CDS**	0.25	0.35	0.25	0	–	–
**RAxML IG**	0.3	0.2	0.3	0.15	0	–
**RAxML Total**	0.25	0.25	0.25	0.1	0.05	0

Supplementary Figures

Comparative analyses of *Mikania* (Asteraceae: Eupatorieae) plastomes and impact of data partitioning and inference methods on phylogenetic relationships

Verônica A. Thode, Caetano T. Oliveira, Benoît Loeuille, Carolina M. Siniscalchi* and José R. Pirani[5]

*** Correspondence:** *carol.siniscalchi@gmail.com*

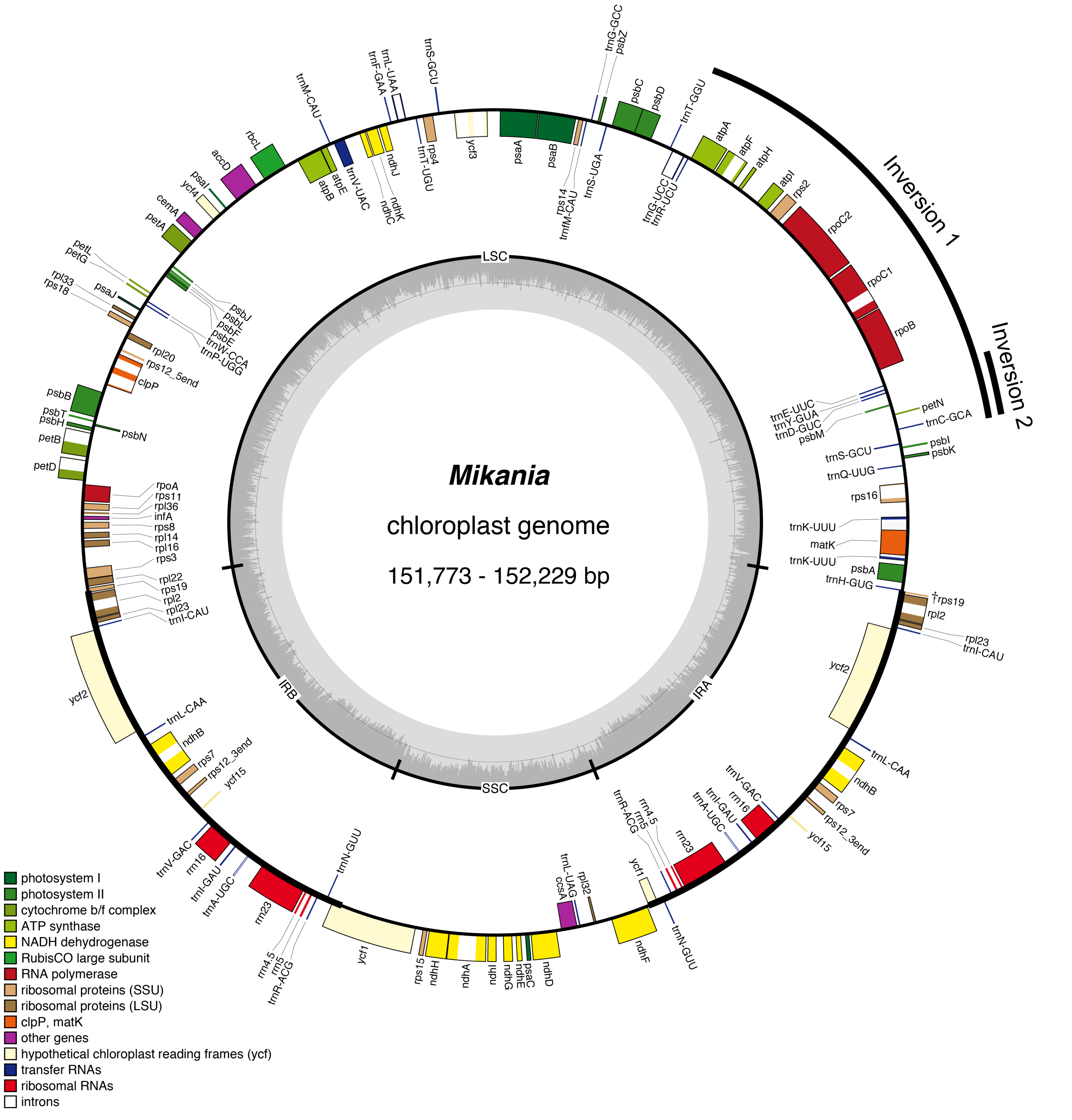

Supplementary Figure 1. Chloroplast genome map of the 19 *Mikania* plastomes assembled in this study. Genes drawn inside the circle are transcribed clockwise, and those drawn outside are transcribed counterclockwise. Genes belonging to different functional groups are colored according to the legend. The darker gray in the inner circle correspond to GC content, while the lighter gray to AT content.

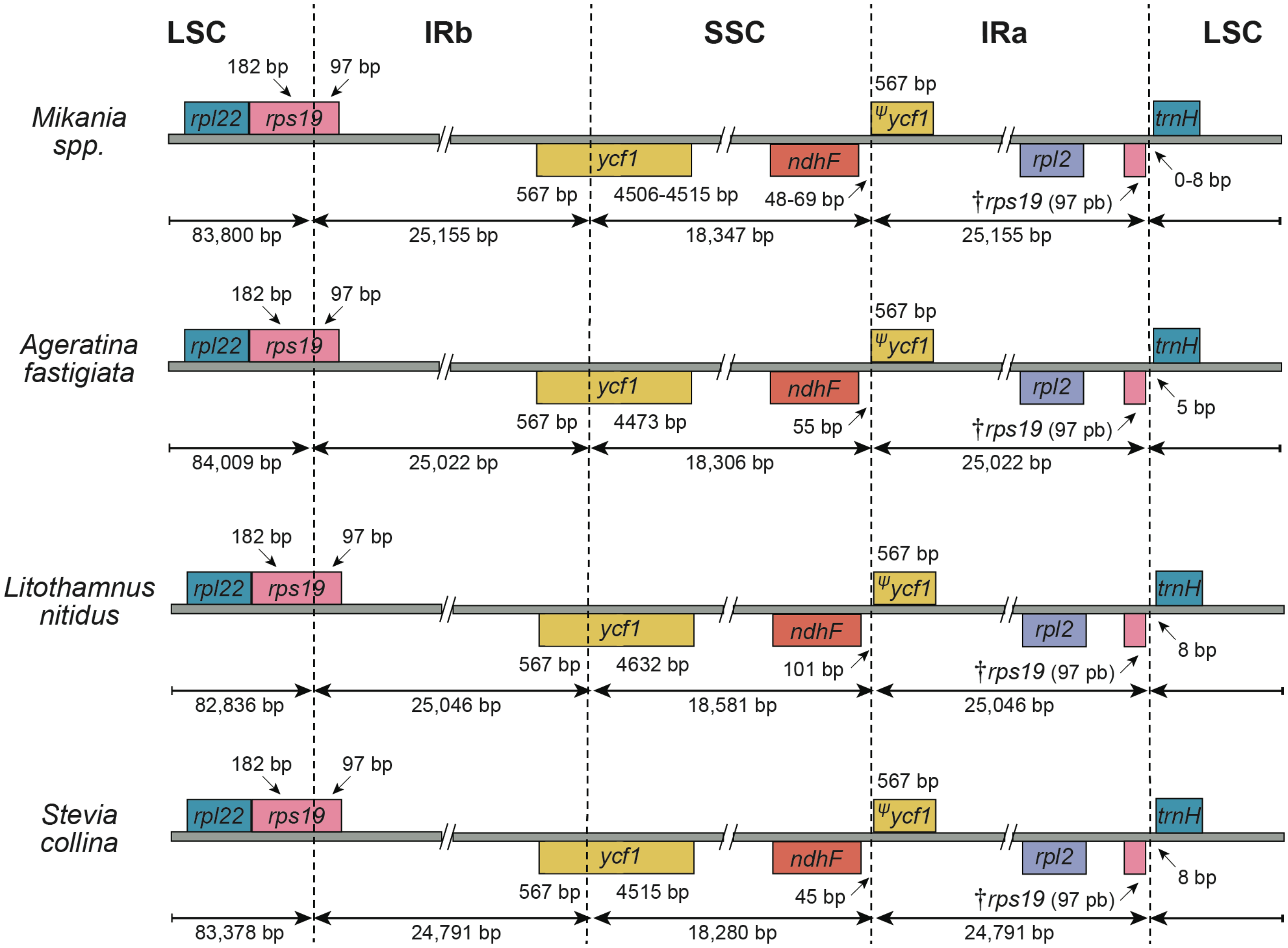

Supplementary Figure 2. Comparisons of the chloroplast genome boundaries between the Large Single Copy (LSC), Inverted Repeat a (IRa), Small Single Copy (SSC), and Inverted Repeat b (IRb) among the *Mikania* plastomes species sequenced here and three other species from tribe Eupatorieae, i.e., *Ageratina fastigiata*, *Litothamnus nitidus,* and *Stevia collina.* Genes shown above the lines are transcribed forward and those below are transcribed reversely. Minimum and maximum sizes for the structures are indicated in base pairs (bp).

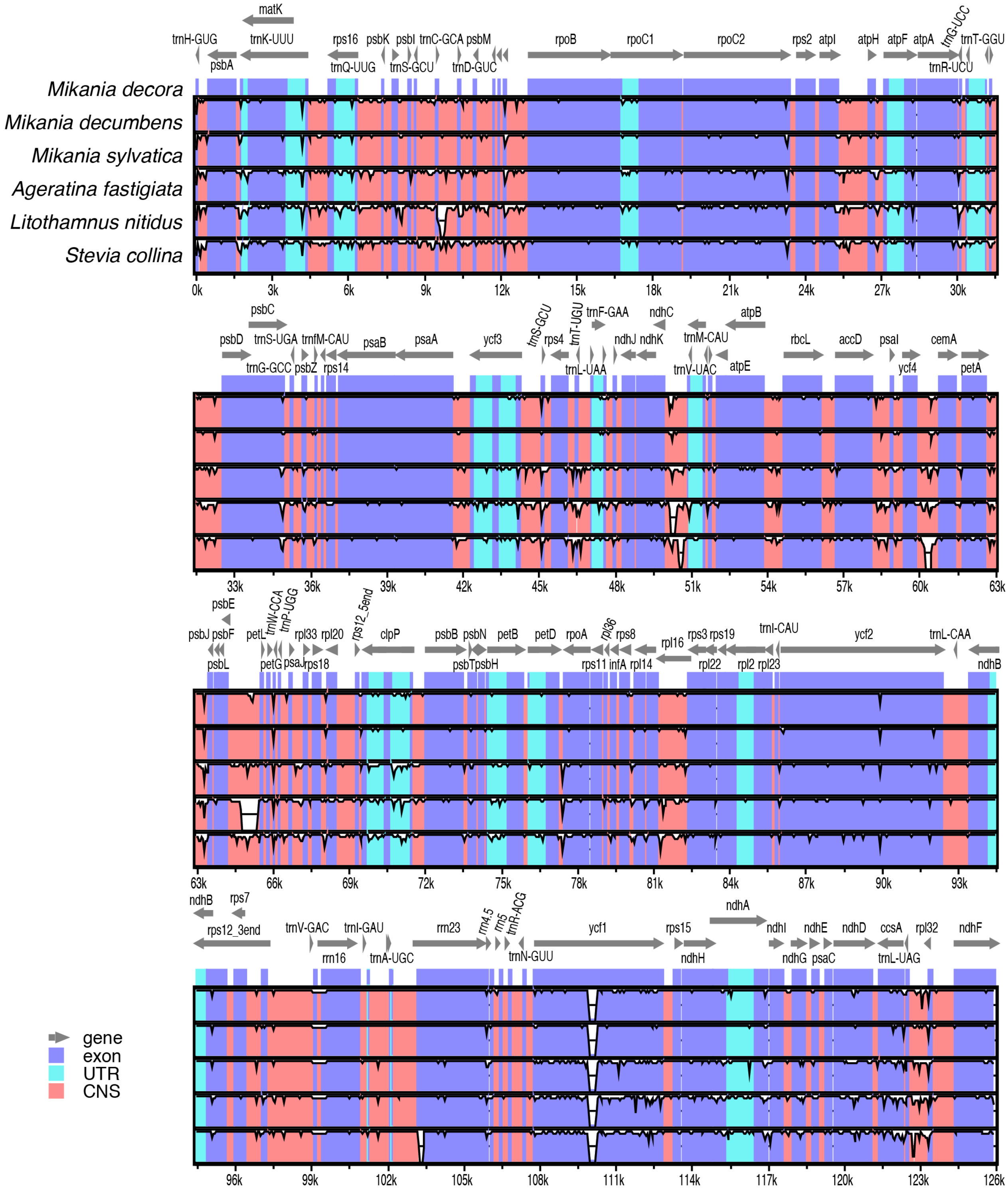

Supplementary Figure 3. Structural alignment using mVISTA among the plastomes of three selected *Mikania* species (i.e., *M. decora*, *M. decumbens*, and *M. syvlatica*), *Ageratina fastigiata*, *Litothamnus nitidus,* and *Stevia collina.* The plastome of *M. decora* was used as reference. Gray arrows indicate the direction of gene transcription. The y-axis represent sequence identity as a percentage between 50 and 100%.

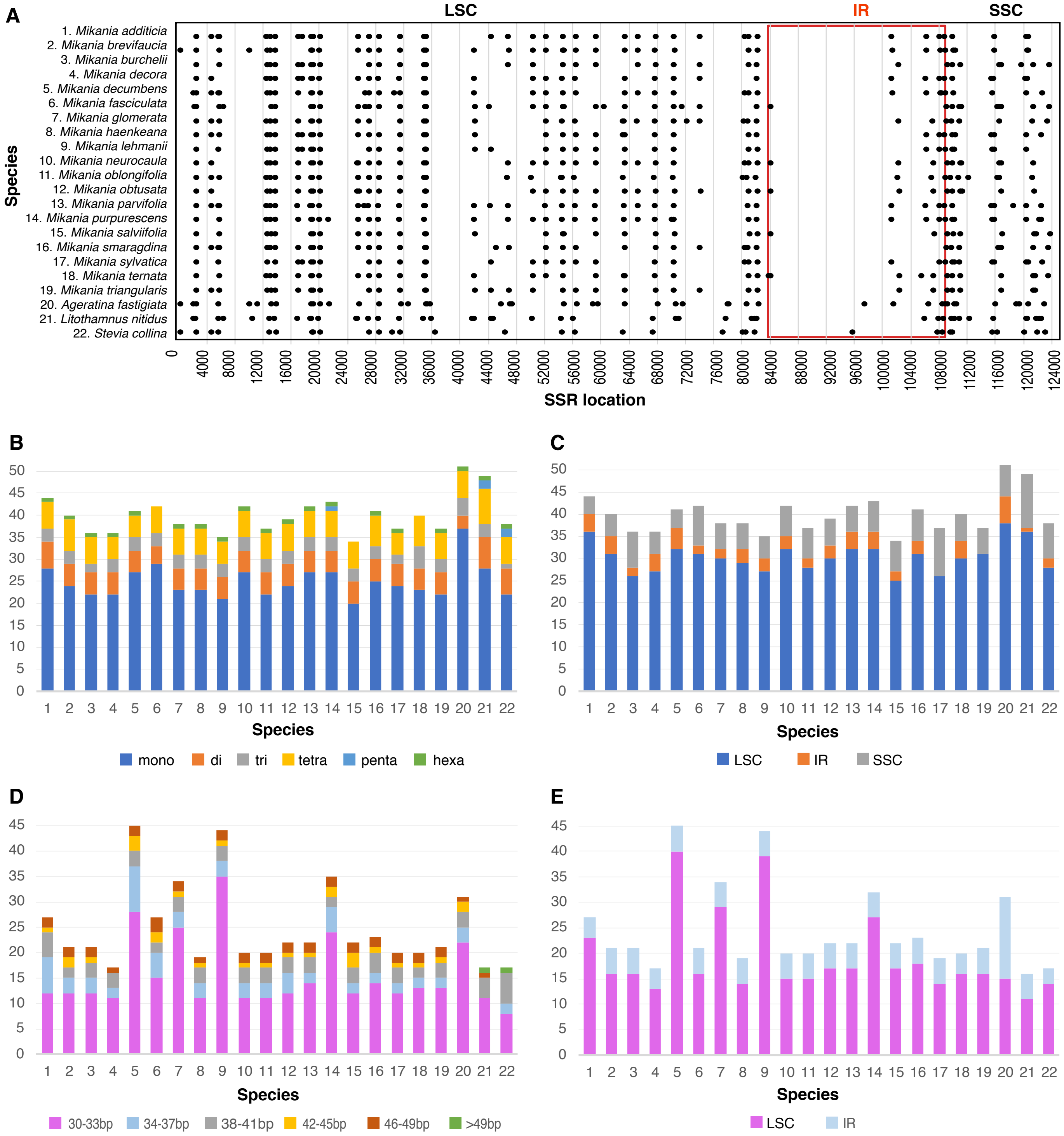

Supplementary Figure 4. (A) Distribution of SSRs in 19 *Mikania* species, *Ageratina fastigiata*, *Litothamnus nitidus*, and *Stevia collina* plastomes assembled in this study. (B) Distribution of SSR types. (C) Distribution of SSRs in the plastome regions. (D) Distribution and length of tandem repeats. (E) Distribution of tandem repeats in genomic regions. Taxa numbers are according to species list in A.

Supplementary Figure 5. Gene discordance analyses. The upper number in each branch indicates the number of concordant gene trees and the lower number the number of conflicting gene trees. Pie charts indicate the proportion of gene trees that support that clade (blue), the proportion that supports the main alternative for that clade (green), the proportion that supports all other topologies (red). (A) Astral topology of the coding dataset. (B) Astral topology of the non-coding dataset

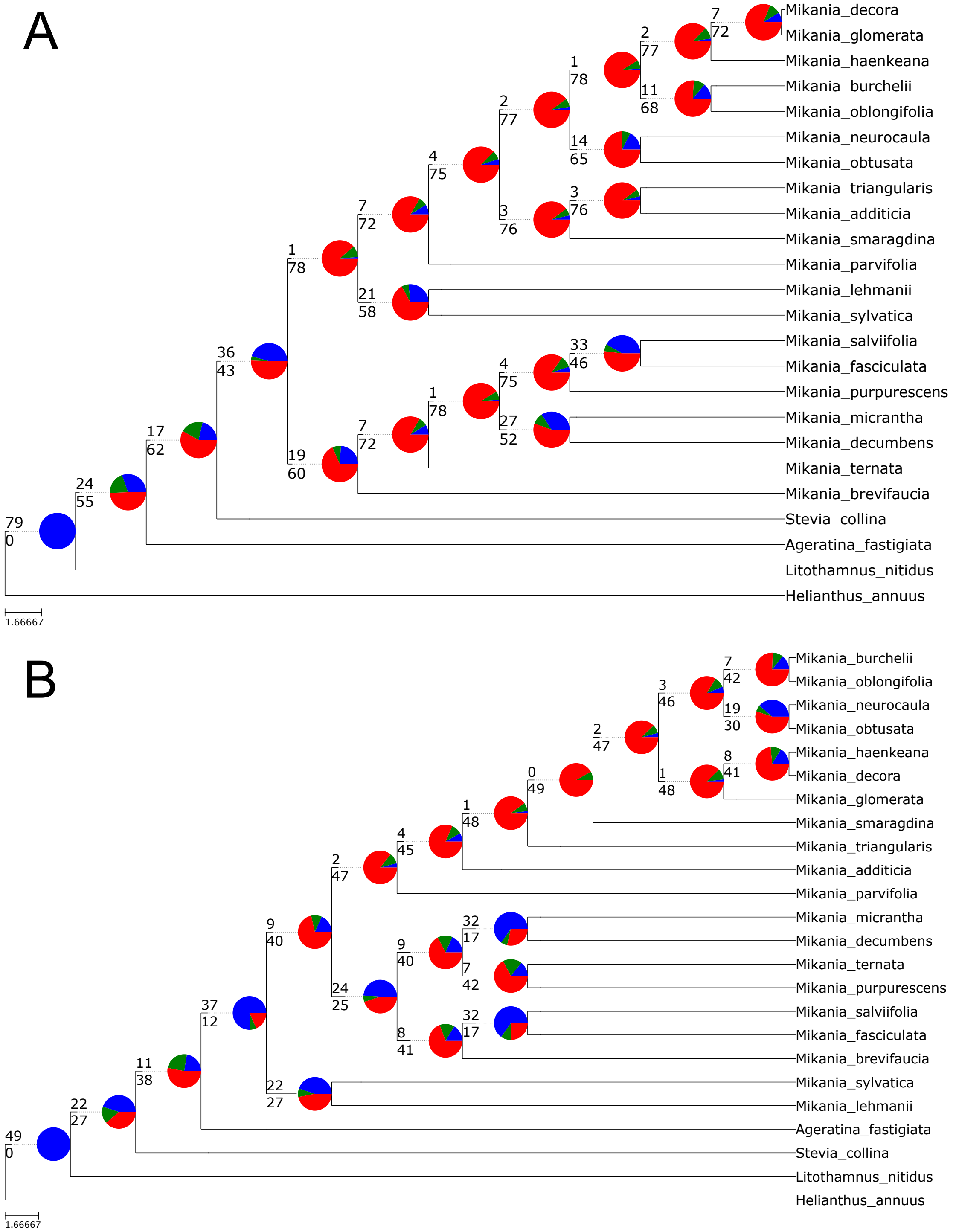

Supplementary Figure 6. Alignment of *ycf1* gene of the species sequenced in this study plus the *ycf1* gene from other Eupatorieae plastomes available in GenBank. The darker regions in the alignment correspond to variations between the sequences and the larger darker regions in *Praxelis clematidea* (NC023833.1) and *Ageratina adenophora* (JF826503.1) inside the red box correspond to an inversion within the *ycf1* gene in these plastomes. *Ageratum* is *Ageratum conyzoides* (MK905238).

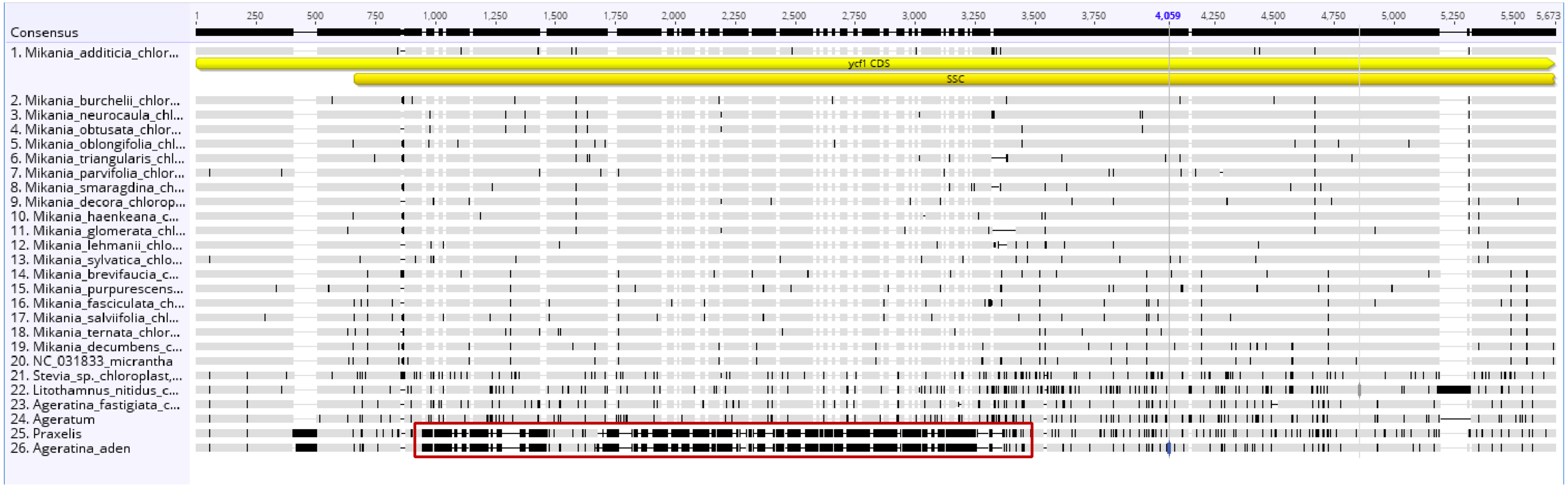

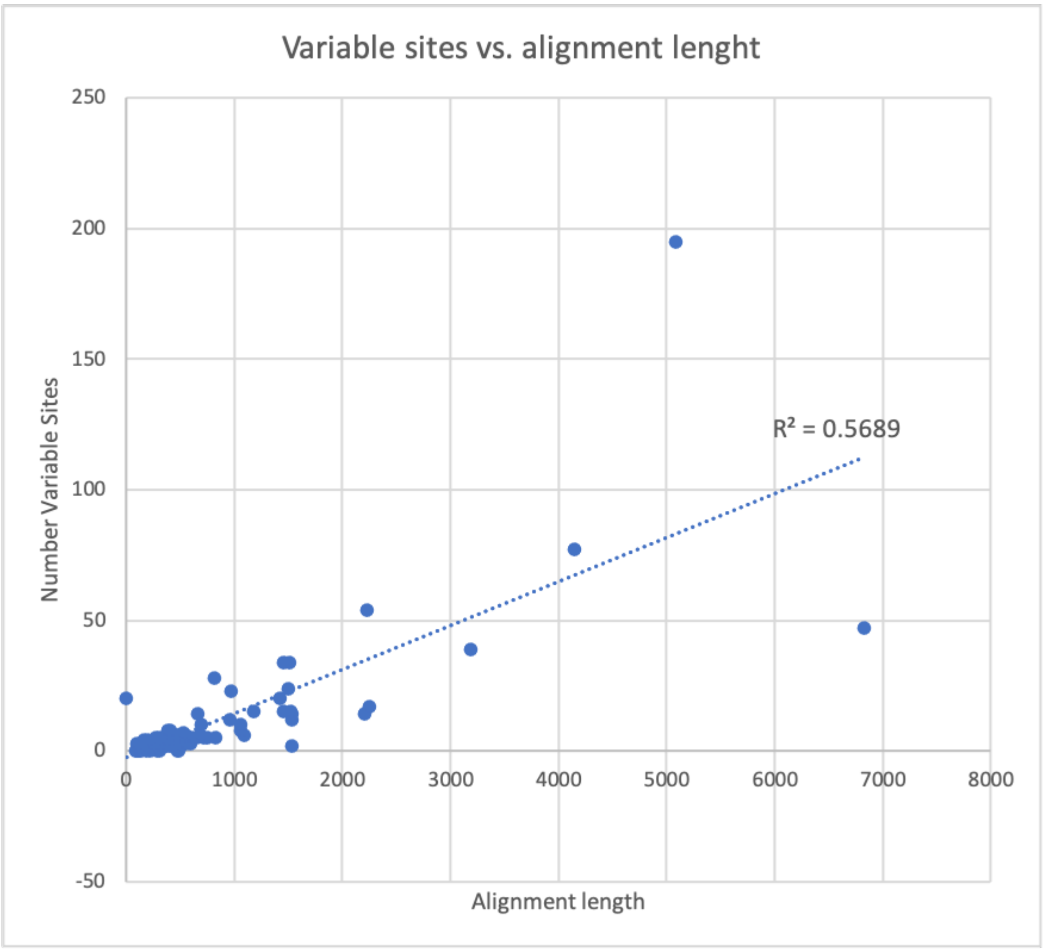

Supplementary Figure 7. Correlation between alignment length and number of variable sites in the coding dataset (CDS). The whole length of each CDS was considered. Pearson correlation coefficient = 0.754260077.
